# 1-[Morpholino(phen­yl)meth­yl]-2-naphthol

**DOI:** 10.1107/S1600536810018064

**Published:** 2010-05-22

**Authors:** Min Min Zhao, Ping Ping Shi

**Affiliations:** a Ordered Matter Science Research Center, College of Chemistry and Chemical, Engineering, Southeast University, Nanjing 211189, People’s Republic of China

## Abstract

There are two independent mol­ecules in the asymmetric unit of the title compound, C_21_H_21_NO_2_, which was synthesized by the one-pot reaction of 2-naphthol, morpholine and benzaldehyde. The dihedral angles between the naphthalene ring systems and the benzene rings are 84.03 (7) and 75.76 (8)° in the two mol­ecules and an intra­molecular O—H⋯N hydrogen bond occurs in each independent mol­ecule.

## Related literature

This backgroud to dielectric ferroelectric phase transition materials, see: Ye *et al.* (2009 ▶); Zhang *et al.* (2009 ▶). For related structures, see: Qu *et al.* (2007 ▶); Li *et al.* (2008 ▶); Wang *et al.* (2009 ▶).
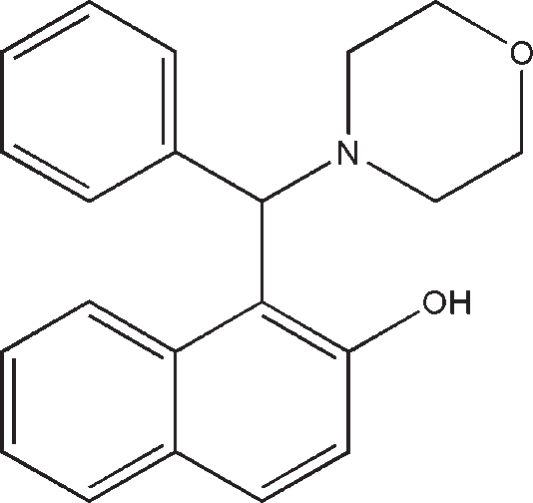


## Experimental

### 

#### Crystal data

C_21_H_21_NO_2_
*M* *_r_* = 319.39
Monoclinic, 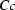

*a* = 10.698 (2) Å
*b* = 19.052 (4) Å
*c* = 16.810 (3) Å
β = 101.13 (3)°
*V* = 3361.5 (12) Å^3^
*Z* = 8
Mo *K*α radiation
μ = 0.08 mm^−1^
*T* = 293 K
0.40 × 0.30 × 0.20 mm

#### Data collection

Rigaku SCXmini diffractometer
Absorption correction: multi-scan (*CrystalClear*; Rigaku, 2005 ▶) *T*_min_ = 0.971, *T*_max_ = 0.984
17285 measured reflections
3849 independent reflections
3058 reflections with *I* > 2σ(*I*)
*R*_int_ = 0.054

#### Refinement

*R*[*F* ^2^ > 2σ(*F* ^2^)] = 0.049
*wR*(*F* ^2^) = 0.118
*S* = 1.08
3849 reflections
433 parameters
2 restraints
H-atom parameters constrained
Δρ_max_ = 0.16 e Å^−3^
Δρ_min_ = −0.17 e Å^−3^

### 

Data collection: *CrystalClear* (Rigaku, 2005 ▶); cell refinement: *CrystalClear*; data reduction: *CrystalClear*; program(s) used to solve structure: *SHELXS97* (Sheldrick, 2008 ▶); program(s) used to refine structure: *SHELXL97* (Sheldrick, 2008 ▶); molecular graphics: *SHELXTL* (Sheldrick, 2008 ▶); software used to prepare material for publication: *PRPKAPPA* (Ferguson, 1999 ▶).

## Supplementary Material

Crystal structure: contains datablocks I, global. DOI: 10.1107/S1600536810018064/jh2156sup1.cif

Structure factors: contains datablocks I. DOI: 10.1107/S1600536810018064/jh2156Isup2.hkl

Additional supplementary materials:  crystallographic information; 3D view; checkCIF report

## Figures and Tables

**Table 1:** Hydrogen-bond geometry (Å, °)

*D*—H⋯*A*	*D*—H	H⋯*A*	*D*⋯*A*	*D*—H⋯*A*
O2—H2*A*⋯N1	0.82	1.92	2.616 (2)	142
O3—H3*A*⋯N2	0.82	1.90	2.603 (3)	143

**Fig. 1.**
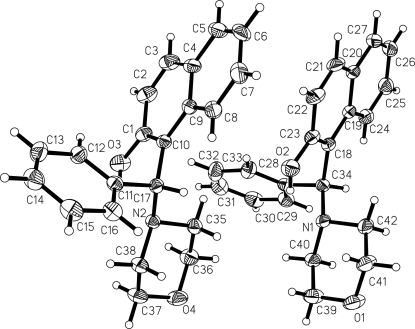
The molecular structure of the title compound, showing the atomic numbering scheme. Displacement ellipsoids are drawn at the 30% probability level.

## supplementary crystallographic information

### Comment

Betti-type reaction is an important method to synthesize chiral ligands and by this method many unnatural compounds derived from naphthalen-2-ol have been obtained (Qu *et al.*, 2007; Li *et al.*, 2008; Wang *et al.*, 2009). Here we report the synthesis and crystal structure of the title compound (Fig. 1), obtained by a three-component condensation reaction of 2-naphthol, benzaldehyde and morpholine under solvent-free condition.

This study is a part of our systematic investigation of dielectric ferroelectric, phase transitions materials (Ye *et al.*, 2009; Zhang *et al.*, 2009) that include metal-organic coordination compounds with organic ligands or are related to the structures with both organic and inorganic building fragments. Below the melting point (m.p. 443 K) of the 1-(morpholino(phenyl)methyl)naphthalen-2-ol, the dielectric constant as a function of temperature goes smoothly, and there is no dielectric anomaly observed (dielectric constant equaling to 3.6 to 5.3, measured temperature ranges: 80 K to 430 K).

In the molecule of the title compound (Fig. 1), the bond lengths and angles are within their normal ranges. There are two molecules which have the same relative conformation in one asymmetric unit. The dihedral angle between the naphthylene ring A (C1–C10) and benzene ring B (C11–C16) is A/B =84.05 °. The dihedral angle between the naphthylene ring C (C18–C27) and benzene ring D (C28–C33) is C/D =75.80 °. The morpholine ring in two molecules both adopt chair conformation. The two molecules are both stabilized by strong intramolecular O—H···N hydrogen bonding(Table 1). The intermolecular attractions are only on the order of Van der Waals forces.

### Experimental

Benzaldehyde (1.06 g, 0.010 mol) was added to 2-naphthol (1.44 g, 0.010 mol) without solvent. Then morpholine (0.87 g, 0.010 mol) was added dropwise with cooling to 0°C to the above mixture. The temperature was raised to 120°C in one hour gradually and the mixture was stirred at this temperature for 10 hours. Then 15 ml of ethanol 95% was added, after heating under reflux for 30 min, the precipitate was filtered off and washed 3 times with a small amount of ethanol 95% to give the title compound. Single crystals suitable for X-ray diffraction analysis were obtained from slow evaporation of chloroform solution.

### Refinement

All H atoms were calculated geometrically, with C—H = 0.93–0.97 Å, O—H= 0.82 Å, and refined as riding with Uiso(H)= 1.2Ueq(C) or 1.2Ueq(O) for hydroxy hydrogen atoms.

### Crystal data

**Table:**

C_21_H_21_NO_2_	*F*(000) = 1360
*M**_r_* = 319.39	*D*_x_ = 1.262 Mg m^−^^3^
Monoclinic, *C**c*	Mo *K*α radiation, λ = 0.71073 Å
Hall symbol: C -2yc	Cell parameters from 15031 reflections
*a* = 10.698 (2) Å	θ = 3.0–27.7°
*b* = 19.052 (4) Å	µ = 0.08 mm^−^^1^
*c* = 16.810 (3) Å	*T* = 293 K
β = 101.13 (3)°	Prism, colorless
*V* = 3361.5 (12) Å^3^	0.40 × 0.30 × 0.20 mm
*Z* = 8	

### Data collection

**Table:**

Rigaku SCXmini diffractometer	3849 independent reflections
Radiation source: fine-focus sealed tube	3058 reflections with *I* > 2σ(*I*)
graphite	*R*_int_ = 0.054
Detector resolution: 13.6612 pixels mm^-1^	θ_max_ = 27.5°, θ_min_ = 3.0°
CCD_Profile_fitting scans	*h* = −13→13
Absorption correction: multi-scan (*CrystalClear*; Rigaku, 2005)	*k* = −24→24
*T*_min_ = 0.971, *T*_max_ = 0.984	*l* = −21→21
17285 measured reflections	

### Refinement

**Table:**

Refinement on *F*^2^	Primary atom site location: structure-invariant direct methods
Least-squares matrix: full	Secondary atom site location: difference Fourier map
*R*[*F*^2^ > 2σ(*F*^2^)] = 0.049	Hydrogen site location: inferred from neighbouring sites
*wR*(*F*^2^) = 0.118	H-atom parameters constrained
*S* = 1.08	*w* = 1/[σ^2^(*F*_o_^2^) + (0.0632*P*)^2^] where *P* = (*F*_o_^2^ + 2*F*_c_^2^)/3
3849 reflections	(Δ/σ)_max_ = 0.001
433 parameters	Δρ_max_ = 0.16 e Å^−^^3^
2 restraints	Δρ_min_ = −0.17 e Å^−^^3^

### Special details

**Table:**

Geometry. All esds (except the esd in the dihedral angle between two l.s. planes) are estimated using the full covariance matrix. The cell esds are taken into account individually in the estimation of esds in distances, angles and torsion angles; correlations between esds in cell parameters are only used when they are defined by crystal symmetry. An approximate (isotropic) treatment of cell esds is used for estimating esds involving l.s. planes.
Refinement. Refinement of *F*^2^ against ALL reflections. The weighted *R*-factor wR and goodness of fit *S* are based on *F*^2^, conventional *R*-factors *R* are based on *F*, with *F* set to zero for negative *F*^2^. The threshold expression of *F*^2^ > σ(*F*^2^) is used only for calculating *R*-factors(gt) etc. and is not relevant to the choice of reflections for refinement. *R*-factors based on *F*^2^ are statistically about twice as large as those based on *F*, and *R*- factors based on ALL data will be even larger.

### Fractional atomic coordinates and isotropic or equivalent isotropic displacement parameters (Å^2^)

**Table:**

	*x*	*y*	*z*	*U*_iso_*/*U*_eq_	
N2	0.53090 (16)	0.19838 (9)	0.19756 (11)	0.0335 (4)	
C17	0.5490 (2)	0.12943 (11)	0.24134 (13)	0.0328 (5)	
H5A	0.5742	0.1395	0.2994	0.039*	
C10	0.42262 (19)	0.08974 (11)	0.22848 (13)	0.0336 (5)	
O4	0.53666 (18)	0.34790 (9)	0.18615 (13)	0.0604 (5)	
C1	0.3388 (2)	0.09325 (12)	0.15454 (15)	0.0412 (6)	
O3	0.35751 (19)	0.13448 (9)	0.09251 (11)	0.0565 (5)	
H3A	0.4235	0.1570	0.1063	0.085*	
C11	0.6534 (2)	0.08461 (11)	0.21689 (13)	0.0351 (5)	
C9	0.3940 (2)	0.04394 (11)	0.28937 (14)	0.0363 (5)	
C4	0.2814 (2)	0.00276 (12)	0.27362 (17)	0.0473 (6)	
C2	0.2279 (2)	0.05124 (14)	0.13943 (18)	0.0544 (7)	
H18A	0.1729	0.0540	0.0894	0.065*	
C16	0.7790 (2)	0.09322 (13)	0.25646 (16)	0.0456 (6)	
H20A	0.7991	0.1263	0.2976	0.055*	
C8	0.4750 (3)	0.03519 (13)	0.36612 (15)	0.0456 (6)	
H23A	0.5511	0.0601	0.3782	0.055*	
C6	0.3308 (3)	−0.04833 (14)	0.4069 (2)	0.0664 (8)	
H26A	0.3100	−0.0779	0.4464	0.080*	
C38	0.6490 (2)	0.23667 (12)	0.19652 (15)	0.0431 (6)	
H28A	0.7049	0.2084	0.1705	0.052*	
H28B	0.6924	0.2462	0.2516	0.052*	
C3	0.2011 (2)	0.00725 (15)	0.1967 (2)	0.0567 (8)	
H29A	0.1284	−0.0206	0.1853	0.068*	
C35	0.4481 (2)	0.24405 (13)	0.23544 (16)	0.0443 (6)	
H31A	0.4892	0.2545	0.2908	0.053*	
H31B	0.3684	0.2202	0.2368	0.053*	
C5	0.2527 (3)	−0.04283 (13)	0.3339 (2)	0.0614 (8)	
H32A	0.1785	−0.0695	0.3231	0.074*	
C36	0.4222 (2)	0.31094 (13)	0.18800 (19)	0.0534 (7)	
H34A	0.3797	0.3002	0.1330	0.064*	
H34B	0.3659	0.3404	0.2124	0.064*	
C7	0.4425 (3)	−0.00951 (14)	0.42262 (18)	0.0606 (8)	
H38A	0.4966	−0.0139	0.4728	0.073*	
C15	0.8748 (2)	0.05256 (15)	0.23472 (18)	0.0556 (7)	
H40A	0.9590	0.0592	0.2606	0.067*	
C12	0.6263 (2)	0.03396 (12)	0.15697 (16)	0.0463 (6)	
H41A	0.5426	0.0272	0.1301	0.056*	
C13	0.7221 (3)	−0.00653 (15)	0.13678 (19)	0.0610 (8)	
H42A	0.7023	−0.0404	0.0965	0.073*	
C14	0.8455 (3)	0.00233 (15)	0.17493 (19)	0.0578 (8)	
H45A	0.9095	−0.0253	0.1608	0.069*	
C37	0.6193 (3)	0.30486 (13)	0.15105 (19)	0.0556 (7)	
H47A	0.6980	0.3300	0.1508	0.067*	
H47B	0.5800	0.2948	0.0953	0.067*	
C19	−0.0234 (2)	0.20900 (12)	0.42768 (14)	0.0375 (5)	
O2	0.31221 (16)	0.20204 (9)	0.40339 (11)	0.0526 (5)	
H2A	0.3118	0.2429	0.3881	0.079*	
C18	0.0949 (2)	0.23186 (11)	0.40893 (14)	0.0370 (5)	
C34	0.1067 (2)	0.30540 (11)	0.37516 (14)	0.0371 (5)	
H10A	0.0480	0.3362	0.3969	0.045*	
C20	−0.0342 (2)	0.13894 (12)	0.45550 (14)	0.0437 (6)	
N1	0.23850 (18)	0.33300 (9)	0.40308 (11)	0.0377 (5)	
C22	0.1834 (3)	0.11606 (13)	0.44640 (17)	0.0514 (7)	
H15A	0.2525	0.0856	0.4526	0.062*	
C28	0.0683 (2)	0.30619 (12)	0.28318 (14)	0.0381 (6)	
C23	0.1955 (2)	0.18532 (12)	0.41921 (15)	0.0428 (6)	
C21	0.0716 (3)	0.09399 (12)	0.46337 (16)	0.0512 (7)	
H21A	0.0644	0.0480	0.4806	0.061*	
C29	−0.0073 (3)	0.35994 (14)	0.24450 (17)	0.0505 (7)	
H22A	−0.0358	0.3949	0.2753	0.061*	
C25	−0.2415 (3)	0.22877 (16)	0.44269 (17)	0.0551 (7)	
H24A	−0.3109	0.2588	0.4391	0.066*	
C24	−0.1317 (2)	0.25283 (14)	0.42093 (15)	0.0461 (6)	
H25A	−0.1286	0.2984	0.4016	0.055*	
C40	0.2622 (2)	0.39992 (13)	0.36510 (16)	0.0455 (6)	
H27A	0.2040	0.4354	0.3777	0.055*	
H27B	0.2476	0.3944	0.3067	0.055*	
O1	0.4239 (2)	0.43231 (10)	0.48085 (12)	0.0670 (6)	
C42	0.2647 (3)	0.34414 (14)	0.49149 (15)	0.0498 (7)	
H33A	0.2516	0.3007	0.5188	0.060*	
H33B	0.2065	0.3790	0.5055	0.060*	
C27	−0.1502 (3)	0.11634 (15)	0.47600 (16)	0.0538 (7)	
H35A	−0.1572	0.0706	0.4939	0.065*	
C39	0.3979 (3)	0.42304 (16)	0.39600 (18)	0.0603 (8)	
H36A	0.4556	0.3881	0.3815	0.072*	
H36B	0.4130	0.4669	0.3701	0.072*	
C33	0.1083 (2)	0.25492 (15)	0.23553 (16)	0.0496 (7)	
H37A	0.1574	0.2178	0.2601	0.060*	
C26	−0.2507 (3)	0.16011 (17)	0.47003 (17)	0.0600 (8)	
H39A	−0.3260	0.1446	0.4841	0.072*	
C41	0.3999 (3)	0.36858 (14)	0.51903 (18)	0.0577 (8)	
H43A	0.4166	0.3753	0.5773	0.069*	
H43B	0.4576	0.3326	0.5068	0.069*	
C30	−0.0413 (3)	0.36241 (16)	0.16068 (19)	0.0608 (8)	
H44A	−0.0928	0.3986	0.1358	0.073*	
C31	0.0009 (3)	0.31159 (18)	0.11421 (18)	0.0652 (9)	
H46A	−0.0213	0.3134	0.0580	0.078*	
C32	0.0764 (3)	0.25798 (19)	0.15184 (18)	0.0637 (8)	
H48A	0.1060	0.2237	0.1208	0.076*	

### Atomic displacement parameters (Å^2^)

**Table:**

	*U*^11^	*U*^22^	*U*^33^	*U*^12^	*U*^13^	*U*^23^
N2	0.0333 (9)	0.0309 (9)	0.0367 (9)	−0.0003 (7)	0.0080 (7)	0.0016 (7)
C17	0.0354 (11)	0.0343 (11)	0.0281 (11)	−0.0001 (9)	0.0049 (9)	0.0003 (9)
C10	0.0316 (10)	0.0290 (10)	0.0413 (12)	0.0003 (8)	0.0096 (9)	0.0005 (9)
O4	0.0618 (11)	0.0321 (9)	0.0881 (14)	−0.0042 (8)	0.0169 (10)	0.0034 (9)
C1	0.0413 (12)	0.0350 (12)	0.0452 (13)	0.0030 (10)	0.0029 (10)	−0.0010 (10)
O3	0.0657 (11)	0.0534 (10)	0.0429 (10)	−0.0086 (9)	−0.0084 (8)	0.0078 (8)
C11	0.0351 (11)	0.0335 (11)	0.0370 (11)	0.0012 (9)	0.0081 (9)	0.0086 (9)
C9	0.0354 (11)	0.0282 (10)	0.0487 (13)	0.0073 (9)	0.0165 (9)	0.0023 (9)
C4	0.0394 (12)	0.0289 (11)	0.0798 (18)	0.0037 (9)	0.0272 (12)	−0.0002 (11)
C2	0.0378 (13)	0.0491 (14)	0.0688 (18)	0.0004 (11)	−0.0082 (12)	−0.0069 (13)
C16	0.0369 (12)	0.0508 (14)	0.0464 (14)	0.0043 (10)	0.0016 (11)	0.0067 (11)
C8	0.0546 (14)	0.0371 (12)	0.0476 (14)	0.0076 (11)	0.0162 (11)	0.0051 (11)
C6	0.0960 (19)	0.0336 (13)	0.0870 (19)	0.0074 (14)	0.0614 (16)	0.0133 (13)
C38	0.0396 (12)	0.0420 (13)	0.0488 (14)	−0.0011 (10)	0.0113 (10)	0.0094 (11)
C3	0.0312 (12)	0.0477 (14)	0.091 (2)	−0.0051 (11)	0.0111 (13)	−0.0089 (15)
C35	0.0429 (12)	0.0393 (12)	0.0549 (14)	0.0017 (10)	0.0194 (11)	−0.0008 (11)
C5	0.0633 (15)	0.0359 (13)	0.098 (2)	−0.0014 (12)	0.0475 (15)	0.0031 (14)
C36	0.0483 (14)	0.0377 (13)	0.0762 (18)	0.0068 (11)	0.0171 (13)	0.0037 (13)
C7	0.0897 (19)	0.0450 (15)	0.0536 (16)	0.0192 (15)	0.0303 (14)	0.0110 (12)
C15	0.0308 (12)	0.0669 (17)	0.0662 (17)	0.0072 (12)	0.0018 (12)	0.0141 (14)
C12	0.0381 (12)	0.0464 (14)	0.0553 (15)	0.0008 (10)	0.0114 (11)	−0.0073 (12)
C13	0.0588 (16)	0.0544 (16)	0.0750 (19)	−0.0010 (13)	0.0261 (14)	−0.0199 (14)
C14	0.0439 (14)	0.0540 (16)	0.0796 (19)	0.0138 (12)	0.0221 (13)	0.0076 (14)
C37	0.0542 (15)	0.0443 (14)	0.0711 (18)	−0.0077 (12)	0.0192 (13)	0.0130 (13)
C19	0.0454 (12)	0.0327 (11)	0.0341 (11)	−0.0067 (9)	0.0070 (9)	−0.0050 (9)
O2	0.0488 (9)	0.0396 (9)	0.0720 (12)	0.0057 (8)	0.0184 (9)	−0.0030 (9)
C18	0.0441 (12)	0.0278 (11)	0.0399 (12)	−0.0003 (9)	0.0103 (10)	−0.0026 (9)
C34	0.0419 (12)	0.0285 (11)	0.0443 (13)	0.0007 (9)	0.0168 (10)	−0.0001 (9)
C20	0.0594 (15)	0.0375 (12)	0.0332 (12)	−0.0132 (11)	0.0068 (11)	−0.0048 (10)
N1	0.0466 (11)	0.0290 (9)	0.0372 (10)	−0.0056 (8)	0.0069 (8)	−0.0016 (8)
C22	0.0602 (16)	0.0291 (12)	0.0616 (17)	0.0050 (11)	0.0040 (13)	−0.0047 (11)
C28	0.0325 (11)	0.0403 (12)	0.0425 (13)	−0.0050 (9)	0.0096 (10)	−0.0017 (10)
C23	0.0494 (14)	0.0325 (12)	0.0471 (14)	0.0001 (10)	0.0112 (11)	−0.0056 (10)
C21	0.0739 (18)	0.0259 (11)	0.0515 (15)	−0.0061 (11)	0.0060 (13)	0.0006 (11)
C29	0.0495 (14)	0.0477 (14)	0.0553 (16)	0.0022 (11)	0.0123 (12)	0.0063 (12)
C25	0.0458 (14)	0.0672 (17)	0.0542 (15)	−0.0057 (13)	0.0146 (12)	−0.0098 (14)
C24	0.0451 (13)	0.0447 (13)	0.0491 (14)	−0.0023 (11)	0.0105 (11)	−0.0039 (11)
C40	0.0554 (14)	0.0362 (12)	0.0452 (14)	−0.0058 (11)	0.0108 (11)	0.0046 (10)
O1	0.0881 (14)	0.0447 (10)	0.0589 (12)	−0.0257 (10)	−0.0086 (10)	0.0034 (9)
C42	0.0725 (17)	0.0361 (13)	0.0408 (14)	−0.0059 (12)	0.0112 (12)	−0.0002 (11)
C27	0.0680 (17)	0.0514 (15)	0.0442 (14)	−0.0234 (13)	0.0164 (13)	−0.0028 (12)
C39	0.0656 (17)	0.0520 (16)	0.0589 (18)	−0.0195 (14)	0.0008 (14)	0.0107 (13)
C33	0.0402 (13)	0.0583 (16)	0.0480 (15)	0.0033 (12)	0.0027 (11)	−0.0083 (12)
C26	0.0582 (16)	0.0734 (19)	0.0533 (16)	−0.0282 (14)	0.0233 (13)	−0.0129 (14)
C41	0.0773 (19)	0.0415 (14)	0.0468 (15)	−0.0146 (13)	−0.0064 (14)	0.0013 (12)
C30	0.0532 (16)	0.0658 (18)	0.0595 (18)	−0.0066 (14)	0.0012 (13)	0.0185 (15)
C31	0.0532 (17)	0.094 (2)	0.0445 (16)	−0.0167 (16)	−0.0007 (13)	0.0018 (16)
C32	0.0526 (16)	0.086 (2)	0.0529 (17)	−0.0050 (15)	0.0097 (13)	−0.0204 (15)

### Geometric parameters (Å, °)

**Table:**

N2—C38	1.462 (3)	C19—C24	1.415 (3)
N2—C35	1.471 (3)	C19—C20	1.426 (3)
N2—C17	1.500 (3)	C19—C18	1.430 (3)
C17—C11	1.524 (3)	O2—C23	1.363 (3)
C17—C10	1.528 (3)	O2—H2A	0.8200
C17—H5A	0.9800	C18—C23	1.380 (3)
C10—C1	1.387 (3)	C18—C34	1.526 (3)
C10—C9	1.422 (3)	C34—N1	1.493 (3)
O4—C37	1.415 (3)	C34—C28	1.521 (3)
O4—C36	1.418 (3)	C34—H10A	0.9800
C1—O3	1.351 (3)	C20—C21	1.405 (4)
C1—C2	1.413 (3)	C20—C27	1.418 (4)
O3—H3A	0.8200	N1—C40	1.470 (3)
C11—C12	1.384 (3)	N1—C42	1.474 (3)
C11—C16	1.390 (3)	C22—C21	1.349 (4)
C9—C8	1.418 (3)	C22—C23	1.411 (3)
C9—C4	1.419 (3)	C22—H15A	0.9300
C4—C3	1.410 (4)	C28—C33	1.382 (4)
C4—C5	1.413 (4)	C28—C29	1.386 (3)
C2—C3	1.348 (4)	C21—H21A	0.9300
C2—H18A	0.9300	C29—C30	1.386 (4)
C16—C15	1.388 (4)	C29—H22A	0.9300
C16—H20A	0.9300	C25—C24	1.374 (4)
C8—C7	1.370 (4)	C25—C26	1.396 (4)
C8—H23A	0.9300	C25—H24A	0.9300
C6—C5	1.348 (4)	C24—H25A	0.9300
C6—C7	1.386 (4)	C40—C39	1.510 (4)
C6—H26A	0.9300	C40—H27A	0.9700
C38—C37	1.510 (3)	C40—H27B	0.9700
C38—H28A	0.9700	O1—C39	1.410 (4)
C38—H28B	0.9700	O1—C41	1.420 (3)
C3—H29A	0.9300	C42—C41	1.505 (4)
C35—C36	1.500 (4)	C42—H33A	0.9700
C35—H31A	0.9700	C42—H33B	0.9700
C35—H31B	0.9700	C27—C26	1.349 (4)
C5—H32A	0.9300	C27—H35A	0.9300
C36—H34A	0.9700	C39—H36A	0.9700
C36—H34B	0.9700	C39—H36B	0.9700
C7—H38A	0.9300	C33—C32	1.383 (4)
C15—C14	1.379 (4)	C33—H37A	0.9300
C15—H40A	0.9300	C26—H39A	0.9300
C12—C13	1.377 (4)	C41—H43A	0.9700
C12—H41A	0.9300	C41—H43B	0.9700
C13—C14	1.363 (4)	C30—C31	1.374 (4)
C13—H42A	0.9300	C30—H44A	0.9300
C14—H45A	0.9300	C31—C32	1.378 (5)
C37—H47A	0.9700	C31—H46A	0.9300
C37—H47B	0.9700	C32—H48A	0.9300
			
C38—N2—C35	107.70 (17)	C24—C19—C20	117.5 (2)
C38—N2—C17	114.36 (16)	C24—C19—C18	123.3 (2)
C35—N2—C17	109.70 (17)	C20—C19—C18	119.2 (2)
N2—C17—C11	112.99 (17)	C23—O2—H2A	109.5
N2—C17—C10	109.69 (16)	C23—C18—C19	118.6 (2)
C11—C17—C10	111.03 (17)	C23—C18—C34	121.2 (2)
N2—C17—H5A	107.6	C19—C18—C34	120.13 (19)
C11—C17—H5A	107.6	N1—C34—C28	111.68 (18)
C10—C17—H5A	107.6	N1—C34—C18	110.33 (17)
C1—C10—C9	118.9 (2)	C28—C34—C18	111.19 (18)
C1—C10—C17	120.3 (2)	N1—C34—H10A	107.8
C9—C10—C17	120.61 (19)	C28—C34—H10A	107.8
C37—O4—C36	109.43 (19)	C18—C34—H10A	107.8
O3—C1—C10	123.6 (2)	C21—C20—C27	121.3 (2)
O3—C1—C2	115.8 (2)	C21—C20—C19	119.1 (2)
C10—C1—C2	120.6 (2)	C27—C20—C19	119.6 (2)
C1—O3—H3A	109.5	C40—N1—C42	107.33 (18)
C12—C11—C16	118.5 (2)	C40—N1—C34	113.63 (17)
C12—C11—C17	121.7 (2)	C42—N1—C34	110.61 (18)
C16—C11—C17	119.7 (2)	C21—C22—C23	120.1 (2)
C8—C9—C4	116.9 (2)	C21—C22—H15A	120.0
C8—C9—C10	123.3 (2)	C23—C22—H15A	120.0
C4—C9—C10	119.8 (2)	C33—C28—C29	117.9 (2)
C3—C4—C5	121.1 (2)	C33—C28—C34	121.9 (2)
C3—C4—C9	118.9 (2)	C29—C28—C34	120.2 (2)
C5—C4—C9	120.0 (2)	O2—C23—C18	123.2 (2)
C3—C2—C1	120.7 (2)	O2—C23—C22	115.3 (2)
C3—C2—H18A	119.6	C18—C23—C22	121.5 (2)
C1—C2—H18A	119.6	C22—C21—C20	121.4 (2)
C15—C16—C11	120.2 (2)	C22—C21—H21A	119.3
C15—C16—H20A	119.9	C20—C21—H21A	119.3
C11—C16—H20A	119.9	C30—C29—C28	121.1 (3)
C7—C8—C9	120.8 (2)	C30—C29—H22A	119.5
C7—C8—H23A	119.6	C28—C29—H22A	119.5
C9—C8—H23A	119.6	C24—C25—C26	121.2 (3)
C5—C6—C7	119.5 (3)	C24—C25—H24A	119.4
C5—C6—H26A	120.3	C26—C25—H24A	119.4
C7—C6—H26A	120.3	C25—C24—C19	120.7 (2)
N2—C38—C37	109.70 (19)	C25—C24—H25A	119.7
N2—C38—H28A	109.7	C19—C24—H25A	119.7
C37—C38—H28A	109.7	N1—C40—C39	109.8 (2)
N2—C38—H28B	109.7	N1—C40—H27A	109.7
C37—C38—H28B	109.7	C39—C40—H27A	109.7
H28A—C38—H28B	108.2	N1—C40—H27B	109.7
C2—C3—C4	121.1 (2)	C39—C40—H27B	109.7
C2—C3—H29A	119.5	H27A—C40—H27B	108.2
C4—C3—H29A	119.5	C39—O1—C41	109.4 (2)
N2—C35—C36	109.7 (2)	N1—C42—C41	109.8 (2)
N2—C35—H31A	109.7	N1—C42—H33A	109.7
C36—C35—H31A	109.7	C41—C42—H33A	109.7
N2—C35—H31B	109.7	N1—C42—H33B	109.7
C36—C35—H31B	109.7	C41—C42—H33B	109.7
H31A—C35—H31B	108.2	H33A—C42—H33B	108.2
C6—C5—C4	121.2 (3)	C26—C27—C20	121.1 (3)
C6—C5—H32A	119.4	C26—C27—H35A	119.4
C4—C5—H32A	119.4	C20—C27—H35A	119.4
O4—C36—C35	111.2 (2)	O1—C39—C40	111.9 (2)
O4—C36—H34A	109.4	O1—C39—H36A	109.2
C35—C36—H34A	109.4	C40—C39—H36A	109.2
O4—C36—H34B	109.4	O1—C39—H36B	109.2
C35—C36—H34B	109.4	C40—C39—H36B	109.2
H34A—C36—H34B	108.0	H36A—C39—H36B	107.9
C8—C7—C6	121.6 (3)	C28—C33—C32	121.1 (3)
C8—C7—H38A	119.2	C28—C33—H37A	119.5
C6—C7—H38A	119.2	C32—C33—H37A	119.5
C14—C15—C16	120.3 (2)	C27—C26—C25	119.8 (3)
C14—C15—H40A	119.9	C27—C26—H39A	120.1
C16—C15—H40A	119.9	C25—C26—H39A	120.1
C13—C12—C11	120.6 (2)	O1—C41—C42	111.9 (2)
C13—C12—H41A	119.7	O1—C41—H43A	109.2
C11—C12—H41A	119.7	C42—C41—H43A	109.2
C14—C13—C12	121.0 (3)	O1—C41—H43B	109.2
C14—C13—H42A	119.5	C42—C41—H43B	109.2
C12—C13—H42A	119.5	H43A—C41—H43B	107.9
C13—C14—C15	119.4 (3)	C31—C30—C29	120.2 (3)
C13—C14—H45A	120.3	C31—C30—H44A	119.9
C15—C14—H45A	120.3	C29—C30—H44A	119.9
O4—C37—C38	112.0 (2)	C30—C31—C32	119.3 (3)
O4—C37—H47A	109.2	C30—C31—H46A	120.4
C38—C37—H47A	109.2	C32—C31—H46A	120.4
O4—C37—H47B	109.2	C31—C32—C33	120.4 (3)
C38—C37—H47B	109.2	C31—C32—H48A	119.8
H47A—C37—H47B	107.9	C33—C32—H48A	119.8
			
C38—N2—C17—C11	−47.7 (2)	C24—C19—C18—C23	178.7 (2)
C35—N2—C17—C11	−168.80 (18)	C20—C19—C18—C23	−0.6 (3)
C38—N2—C17—C10	−172.15 (18)	C24—C19—C18—C34	−3.7 (3)
C35—N2—C17—C10	66.7 (2)	C20—C19—C18—C34	177.0 (2)
N2—C17—C10—C1	36.7 (3)	C23—C18—C34—N1	−34.7 (3)
C11—C17—C10—C1	−88.9 (2)	C19—C18—C34—N1	147.7 (2)
N2—C17—C10—C9	−148.84 (18)	C23—C18—C34—C28	89.8 (3)
C11—C17—C10—C9	85.6 (2)	C19—C18—C34—C28	−87.8 (2)
C9—C10—C1—O3	−179.4 (2)	C24—C19—C20—C21	−179.7 (2)
C17—C10—C1—O3	−4.8 (3)	C18—C19—C20—C21	−0.3 (3)
C9—C10—C1—C2	−1.0 (3)	C24—C19—C20—C27	−0.8 (3)
C17—C10—C1—C2	173.6 (2)	C18—C19—C20—C27	178.6 (2)
N2—C17—C11—C12	−94.3 (2)	C28—C34—N1—C40	48.6 (3)
C10—C17—C11—C12	29.4 (3)	C18—C34—N1—C40	172.83 (19)
N2—C17—C11—C16	87.1 (2)	C28—C34—N1—C42	169.42 (19)
C10—C17—C11—C16	−149.2 (2)	C18—C34—N1—C42	−66.4 (2)
C1—C10—C9—C8	178.3 (2)	N1—C34—C28—C33	79.5 (3)
C17—C10—C9—C8	3.8 (3)	C18—C34—C28—C33	−44.2 (3)
C1—C10—C9—C4	0.2 (3)	N1—C34—C28—C29	−99.4 (2)
C17—C10—C9—C4	−174.4 (2)	C18—C34—C28—C29	136.8 (2)
C8—C9—C4—C3	−177.0 (2)	C19—C18—C23—O2	−179.7 (2)
C10—C9—C4—C3	1.3 (3)	C34—C18—C23—O2	2.6 (4)
C8—C9—C4—C5	1.7 (3)	C19—C18—C23—C22	1.0 (4)
C10—C9—C4—C5	180.0 (2)	C34—C18—C23—C22	−176.7 (2)
O3—C1—C2—C3	178.9 (2)	C21—C22—C23—O2	−179.6 (2)
C10—C1—C2—C3	0.4 (4)	C21—C22—C23—C18	−0.3 (4)
C12—C11—C16—C15	1.2 (4)	C23—C22—C21—C20	−0.8 (4)
C17—C11—C16—C15	179.9 (2)	C27—C20—C21—C22	−177.8 (2)
C4—C9—C8—C7	−2.0 (3)	C19—C20—C21—C22	1.1 (4)
C10—C9—C8—C7	179.8 (2)	C33—C28—C29—C30	−0.3 (4)
C35—N2—C38—C37	−57.8 (3)	C34—C28—C29—C30	178.7 (2)
C17—N2—C38—C37	180.0 (2)	C26—C25—C24—C19	−1.7 (4)
C1—C2—C3—C4	1.1 (4)	C20—C19—C24—C25	1.7 (3)
C5—C4—C3—C2	179.4 (2)	C18—C19—C24—C25	−177.6 (2)
C9—C4—C3—C2	−1.9 (4)	C42—N1—C40—C39	58.2 (3)
C38—N2—C35—C36	59.0 (2)	C34—N1—C40—C39	−179.2 (2)
C17—N2—C35—C36	−175.91 (19)	C40—N1—C42—C41	−58.3 (3)
C7—C6—C5—C4	−1.1 (4)	C34—N1—C42—C41	177.25 (19)
C3—C4—C5—C6	178.5 (3)	C21—C20—C27—C26	178.6 (3)
C9—C4—C5—C6	−0.2 (4)	C19—C20—C27—C26	−0.3 (4)
C37—O4—C36—C35	58.7 (3)	C41—O1—C39—C40	58.0 (3)
N2—C35—C36—O4	−60.5 (3)	N1—C40—C39—O1	−59.7 (3)
C9—C8—C7—C6	0.8 (4)	C29—C28—C33—C32	1.5 (4)
C5—C6—C7—C8	0.8 (4)	C34—C28—C33—C32	−177.5 (2)
C11—C16—C15—C14	−1.4 (4)	C20—C27—C26—C25	0.5 (4)
C16—C11—C12—C13	−0.5 (4)	C24—C25—C26—C27	0.5 (4)
C17—C11—C12—C13	−179.1 (2)	C39—O1—C41—C42	−58.0 (3)
C11—C12—C13—C14	−0.2 (4)	N1—C42—C41—O1	59.5 (3)
C12—C13—C14—C15	0.1 (5)	C28—C29—C30—C31	−0.6 (4)
C16—C15—C14—C13	0.7 (4)	C29—C30—C31—C32	0.5 (4)
C36—O4—C37—C38	−57.9 (3)	C30—C31—C32—C33	0.7 (5)
N2—C38—C37—O4	58.7 (3)	C28—C33—C32—C31	−1.7 (4)

### Hydrogen-bond geometry (Å, °)

**Table:**

*D*—H···*A*	*D*—H	H···*A*	*D*···*A*	*D*—H···*A*
O2—H2A···N1	0.82	1.92	2.616 (2)	142
O3—H3A···N2	0.82	1.90	2.603 (3)	143
